# Dysfunction of natural killer cells in end-stage kidney disease on hemodialysis

**DOI:** 10.1186/s41100-021-00324-0

**Published:** 2021-02-12

**Authors:** Kei Nagai

**Affiliations:** Department of Nephrology, Faculty of Medicine, University of Tsukuba, Tsukuba, Japan

**Keywords:** Natural killer cells, Hemodialysis, Chronic kidney disease, Oxidative stress

## Abstract

Natural killer (NK) cells are known to play an important role in defense against infection and tumors. Although there is no clear consensus, most studies have shown that the number and cytotoxicity of NK cells decreases in end-stage kidney disease (ESKD) patients undergoing hemodialysis. Uremic patients chronically suffer from oxidative stress, which could be responsible for downregulation of the activating receptors on NK cells and modulation of ligand expression for activating receptors. Theoretically, the reduced number of NK cells and decreased function might increase susceptibility to viral infections and cancer development in patients with ESKD. There is emerging evidence that NK cell numbers may be an outcome predictor in renal transplantation; however, the clinical significance of NK cell dysfunction in dialysis patients requires clarification. In this review, I describe NK cell number, cytotoxic activity, and activating mechanisms in the context of uremia and oxidative stress, which is anticipated to assist in elucidating the mechanisms underlying immunodeficiency in dialysis patients.

## Background

Loss of renal function, inflammation, and oxidative stress occur in end-stage kidney disease (ESKD) patients. These patients are highly susceptible to infections [1] and have an increased risk of virus-associated cancers [2]. Retention of uremic toxins and cytokines is key mechanisms that underlie the generation of oxidative stress and inflammation [3, 4]. Many clinical studies have focused on such immune system alterations in patients with ESKD [5, 6].

Natural killer (NK) cells are present in the bloodstream, spleen, and in certain non-lymphoid organs, and are known to play an important defensive role in innate immunity against infection and tumors [7, 8]. Some studies have shown that NK cell cytotoxicity is decreased in hemodialysis (HD) patients [9–11]. However, there is little clinically relevant evidence for the involvement of these cells in ESKD patients. Therefore, this review aims to summarize the dysfunction of NK cells in the uremic milieu and under dialysis conditions, as well as its clinical implications. I anticipate that this review will assist in elucidating aspects of the mechanism of immunodeficiency in ESKD undergoing dialysis.

## The role of natural killer cells and clinical implications

**Fig. 1:**
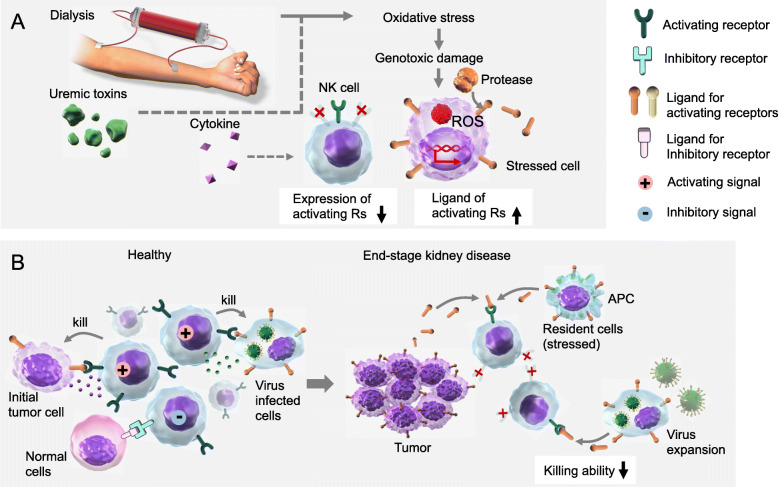
NK cell receptor expression and function in end-stage kidney disease and chronic hemodialysis. **a** Uremic patients suffer from excessive inflammatory cytokine and oxidative stress concurrent with maintenance HD. Chronic exposure to oxidative stress could be responsible for downregulation of the activating receptors on NK cells. Various types of stress, including oxidative stress-induced genotoxic stress, modulate expression of ligands for NK activating receptors. Moreover, surface expression levels of membrane ligands on stressed cells can be finely tuned by the regulation of their release in soluble form by various processes including protease-mediated cleavage. **b** To achieve appropriate effector function, NK cells must recognize and distinguish healthy “self” cells from abnormal “non-self” cells via receptor-ligand interactions at the cell surface. Generally, the activating receptors recognize ligands on stressed cells such as tumor cells and virally infected cells, while the inhibitory receptors recognize healthy normal cells. Soluble forms of ligands can cause downregulation of surface expression of activating receptors on NK cells by promoting their internalization and degradation, leading to reduced immune responses against tumors and virally infected cells. As a result, end-stage kidney disease patients undergoing chronic dialysis are expected to be prone to tumor formation and viral expansion

The term “natural killer” was derived from the ability of these cells to kill target cells without the need for clonal expansion and differentiation, which is required for the effector responses of other killer cells such as cytotoxic T lymphocytes. To achieve appropriate effector function, NK cells must recognize and distinguish healthy “self” cells from abnormal “non-self” cells via receptor-ligand interactions at the plasma membrane. The receptors generate activating or inhibitory signals that promote or inhibit NK responses, respectively. Generally, the activating receptors recognize ligands on injured and stressed cells, and the inhibitory receptors recognize healthy normal cells (Fig. 1a and b).

Activating receptors on NK cells include natural killer group 2 member D (NKG2D), cluster of differentiation (CD) 226, CD16, and natural cytotoxic receptors (NCRs), which include natural killer cell p30-related protein (NKp30), NKp44, and NKp46 [8, 12]. They have common and unique features, and the lack of clonotypic receptors can be compensated for by multiple NK cell activation receptors. Among them, NKG2D is a homodimer forming a C-type lectin-like type II transmembrane receptor that is highly conserved from mouse to human and is well characterized [13]. NKG2D is constitutively expressed on NK cells and recognizes stress-inducible ligands that are structurally related to self-major histocompatibility complex (MHC)-I including MHC class I chain-related gene A (MICA) and B (MICB) [14]. These ligands are found on virally infected cells and tumor cells but not on normal cells. After ligand binding to the NK cell receptors, polypeptide chains containing immunoreceptor tyrosine-based activation motifs (ITAM) are recruited and associate to transmit activating signals [8]. As a result, cytoplasmic granules released from NK cells kill cells considered as non-self. Granule proteins such as perforin and granzymes initiate a sequence of signaling events that cause the death of target cells by apoptosis. Another function is through interferon-γ secretion by NK cells to activate macrophages and increase the capacity of macrophages to kill phagocytosed bacteria [12].

As NK cells have such effector functions, they play several important roles in defense against intracellular microbes. While NK cell congenital deficiency is rare, it results in susceptibility to herpes virus and papillomavirus infections [15]. In the context of cancer, NK cells can detect reduced levels of surface expression of self-MHC-I molecules to identify autologous cells that have undergone malignant transformation [7]. This “missing-self” state induces loss of inhibitory signals and results in NK cell activation as anti-tumor immunity. Moreover, transformed cells express increased numbers of stress-induced molecules on their surface that can be recognized by specific NK cell receptors. Numerous studies in rodents have demonstrated the importance of NK cells in tumor clearance [16, 17].

## NK cell number and dysfunction in end-stage kidney disease with HD

The NK cell compartment, identified by flow cytometry as CD56^+^CD16^+^CD3^−^ cells, represents several percent of peripheral blood lymphocytes. There are studies showing the characteristics of NK cells in HD patients; however, they have led to conflicting results in HD patients partially because of limited study size [11, 18–20]. The following discussion focuses on NK cell number, cytotoxic function, and activating mechanisms.

As age and gender affect NK cell count [21], multivariate analysis should be utilized to examine the relationship between NK cell number and residual renal function in ESKD patients. In contrast to other lymphocyte subsets, decreased creatinine clearance was the only factor found to confound lowered NK cell counts in ESKD patients without dialysis therapy, even when other factors were considered in multivariate analysis [22]. In contrast, in a study of patients with chronic kidney disease (CKD) G4 or better, presentation of mild uremia had a predominant effect on NK cells by increasing NK cell percentage [23]. This discrepancy led us to speculate that uremia may dramatically alter the lymphocyte fraction, depending on its severity.

Most studies have shown that NK cell cytotoxicity is decreased in HD patients [9–11, 20, 24]. However, these findings must be carefully interpreted because lowered NK cell populations are occasionally associated with lowered NK cell cytotoxicity. In a cohort study of 41 HD patients and 31 healthy controls, NK cell function was similar in subgroups of HD and healthy controls matched for NK cell counts [22]. Nevertheless, the effect of uremic serum on samples of healthy donor NK cells in vitro indicated that uremic factors might decrease NK cell cytotoxicity [10].

To gain the appropriate NK cell cytotoxic function, activation proceeds through activating receptors on NK cells [8]. NK cell-activating CD16 and NCRs transduce signaling via zeta-chain bearing ITAM. Phosphorylation of the zeta-chain is an early event that follows the triggering of these receptors [25]. The NK cell zeta-chain is downregulated in patients with cancer due to chronic inflammation involving Toll-like receptor-mediated signaling [25, 26]. Furthermore, chronic inflammation could be responsible for NK cell zeta-chain downregulation in HD patients, contributing to decreased NK cell activity [24]. Another report showed reduced expression of the pivotal activating receptor NKG2D on NK cells from HD patients [27]. These studies provide support for the molecular mechanisms of decreased NK cell activity in HD patients [25, 27].

Focusing on HD conditions, previous studies reported an interesting relationship between the biocompatibility of the HD membrane and NK cell activity. HD with cuprophan membranes elicits a higher proportion of CD3^−^CD56^+^ NK cells and a decrease in their cytotoxicity compared with the control group [11, 19]. This alteration was related to the use of cuprophan membranes, since biocompatible membranes, such as polyacrylonitrile and polysulfone, did not significantly affect NK cells [11]. These findings support the notion that the use of bioincompatible membranes induces an activation state in NK cells of ESKD patients undergoing HD. Such pre-activated innate immune cells can be a factor in the prevailing systemic oxidative stress, inflammation, and tissue damage [24–26].

The general biology of NK cells has been elucidated with the development of technologies such as flow cytometry, and the properties of NK cells in ESKD patients have been reported over the past three decades in a modest number of reports. However, the conclusions drawn from the characterization of NK cells in ESKD patients remain inconsistent, possibly due to differences in patient cohort properties and analytical methods as summarized in Table 1. Therefore, further research is required to clarify the molecular mechanisms of alterations in NK cell number and function in the context of uremia and dialysis therapy, by using larger cohorts and more consistent analytical methodologies.

**Table 1:** Review of NK cell function in end-stage kidney disease

Summary				Study design	Results and interpretation	Ref., year
	C↓			Comparison between three dialyzer membranes of different composition on suppressing NK activity.	Biocompatibility of hemodialyzer membrane affects the significance in impairment of human NK cell cytotoxic function.	[9], 1987
	C↓			Comparing NK cell activity of PBMC between maintenance HD patients and healthy control.	NK cell cytotoxic activity in patients on HD was two thirds of healthy control. Although urea did not suppress the NK cell activity, the guanidino compound did significantly.	[10], 1988
N↓	C↓			Comparing cell activity of NK cell number among 20 undialyzed ESKD patients, 25 patients on HD patients, patients on PD, and healthy control. NK cells were morphologically determined as large granular cells.	In groups of ESKD patients, HD and PD, NK cell number and activity were reduced to less than half of the control group. Particularly, HD patients on cuprophane membrane have an additional negative effect on NK cells.	[20], 1990
N↑	C↓			A prospective study of 8 patients on HD using cuprophane membrane for 2 weeks. NK cells were analyzed by flow cytometry.	After exposure of cuprophane membrane, CD56^+^ NK cells increased from 28 to 59% in peripheral blood lymphocytes and decreased cytotoxicity from 29 to 12%.	[19], 1993
N↑	C↓			Counting peripheral NK cells (CD3^−^CD56^+^ and CD3^−^CD16^+^) in 35 patients on HD.	HD elicits higher proportions of NK cells and decrease in their cytotoxic activity compared with the control group.	[11], 1996
N↑				Lymphocyte subpopulation analysis in 34 patients on HD and 37 patients on PD	The HD and CAPD patients showed increased percentages of NK cells (CD3^−^CD16^+^CD56^+^).	[18], 2005
N↓	C→	R→		Phenotyping NK cells in 210 patients on HD, undialyzed uremic control and healthy volunteers. Functional assay was performed with matching for NK cell count. NKp30, NKp46, NKG2D, and CD226 were examined as activating NK receptors.	In undialyzed patients, lowered NK cell counts were associated with reduced renal clearance. NK counts in HD patients were lower than in healthy control. NK cell functions were similar in subgroups of HD and healthy control. The expression of receptors modulating NK cytotoxicity was not altered.	[22], 2008
N→	C↓			Functional analysis of CD3^+^CD16^+^ NK cells from 33 HD patients and 30 healthy donors. Expression level of CD3 zeta-chain in NK cells was evaluated.	NK cell counts did not differ between HD patients and healthy donors. NK cell zeta-chain downregulation in HD patients seems to contribute to the decreased NK cell activity.	[24], 2008
N→	C↓	R↓	L↑	Comprehensive characterization of NK cell number and cytotoxic function in 66 ESKD patients (59 patients on HD and 7 patients on PD), and experiments to show the function of NK receptors and their ligands in uremic status.	While total NK cell proportion was comparable, patients on chronic dialysis showed a profound decrease in NKG2D^+^ NK cells comparing to healthy donors (56% vs 39%). Combined with increased expression soluble ligands for NKG2D in uremia, NKG2D-mediated NK cytotoxic function was impaired in ESKD patients.	[27], 2009
N↑↓				Lymphocyte subset in peripheral blood from 472 CKD patients (G1, *n*=88; G2 *n*=93; G3, *n*=88; G4, *n*=68; *n*=135) was examined.	Proportion of CD16^+^CD56^+^ NK cells was 9.2 (G1), 10.8 (G2), 12.9 (G3), 13.5 (G4), and 12.8% (G5) in CKD. On contrary, absolute lymphocyte count was substantially decreased from 2.04 (G1) to 1.48 × 10^9^/L (G5).	[23], 2016

*N* Number of natural killer (NK) cells, *C* Cytotoxic function of NK cells, *R* Expression of NK receptors, *L* Ligand expression of activating receptors, *ESKD* End-stage kidney disease, *PBMC* Peripheral blood mononuclear cells, *HD* Hemodialysis, *PD* Peritoneal dialysis, *NKp30*, *-p40* Natural killer cell p30-related protein, p-46 related protein, *NKG2D* Natural killer group 2 member D

## Oxidative stress and expression of activating receptors and their ligands

Dialysis patients and uremic patients chronically suffer from oxidative stress. Recent studies have shown the putative association between characteristics of NK cells, oxidative stress, and uremia (Fig. 1a). Chronic exposure to oxidative stress could be responsible for downregulation of the NK cell zeta-chain in HD patients [24]. Similarly, patients undergoing chronic dialysis showed a profound decrease in NKG2D^+^ NK cells in peripheral blood compared to healthy donors [27]. Uremic serum in vitro could reduce NKG2D expression on NK cells from healthy donors. To directly evaluate the role of reactive oxygen species (ROS) in the downregulation of NKG2D on NK cells from ESKD patients, NK cells were cultivated in the presence of catalase, an enzyme that breaks down H_2_O_2_. Whereas catalase had no effect on NK cells incubated with control serum, this enzyme significantly reversed the ability of serum from ESKD patients to reduce NKG2D expression on NK cells [27]. In the context of these results, it was concluded that ROS is likely to be a central factor in the modulation of NKG2D signal-mediated activation of NK cells in ESKD [27].

However, there are conflicting results showing that NK cell function and expression of receptors modulating NK cytotoxicity, including CD69, NKG2D, and NKp44, were not modified in patients with ESKD and in healthy age-matched controls [22]. Regarding the control mechanism of NK cell function by NKG2D, it appears that humoral factors and cell-extrinsic mechanisms have a critical influence, as well as alteration of the NK cell itself in the uremic milieu. Therefore, ligand expression and cell-extrinsic regulation of NK cells should be carefully examined.

Expression of NKG2D ligands can be induced by various types of stress, such as genotoxicity, infection, heat shock, and oxidative stress [28] (Fig. 1a). At the same time, surface expression levels of membrane NKG2D ligands can be finely tuned by mechanisms implicated in the regulation of its release in soluble form by various processes, including protease-mediated cleavage [29]. Representative NKG2D ligands, MICA and MICB, are cleaved by a protease belonging to the matrix metalloproteinases that undergo modulation of its activity and expression [29]. Generally, soluble forms of MICA can cause the downregulation of surface expression of NKG2D by promoting its internalization and degradation, leading to reduced immune responses against tumors and virally infected cells [30] (Fig. 1b). If not appropriately controlled, ROS can cause severe damage to cellular macromolecules, especially DNA, and promote transcriptional modulation [31–33]. ROS trigger the up-regulation of MICA [34] and have been recently implicated in the downregulation of NKG2D in NK cells [10, 27, 35].

In summary, one of the putative mechanisms of innate immune dysfunction in uremic patients is the reduced activity of NK cells, mainly caused by reduced activation signals due to oxidative stress in ESKD and the HD milieu. Impaired NK cell function results in tumor cells and virally infected cells escaping being killed (Fig. 1b). As a consequence, HD patients are expected to be prone to tumor formation and viral expansion, as shown by clinical-epidemiological studies [2, 36].

## Clinical significance of NK cell dysfunction in end-stage kidney disease has yet to be explained

Patients with primary NK cell deficiency are susceptible to virally driven malignancies [15, 37]. Theoretically, the reduced number of NK cells and possible decreased function might increase susceptibility to viral infections in patients with ESKD, resulting from decreased killing of infected cells and transformed cells [5]. This abrogation of immunological surveillance is clinically apparent because of the increased relative risk of known virally associated tumors, such as genital cancer and tumors associated with human papillomavirus and Epstein-Barr virus [2, 5, 38]. Currently, there is little evidence of a direct association between NK cell dysfunction and clinical outcomes of HD patients including viral infection and cancer.

In contrast, there is emerging evidence that NK cell number may be a predictor of complications in renal transplantation [39–41]. Transplantation patients with both CKD and immunosuppressive therapy show dose-dependent inhibition of NK cell function [42]. Recently, Dendle et al. showed that dysfunction of NK cytotoxicity, but not NK cell number, is associated with the occurrence of infectious complications in renal transplantation [43]. In regard to cancer, reduced NK cell number after renal transplantation in patients who had a previous squamous cell carcinoma (SCC) was associated with increased risk of developing new SCC [44]. As the use of immunosuppressive drugs is closely involved in reducing NK cell numbers [45], it remains difficult to clarify whether changes in NK cell function due to renal dysfunction and dialysis therapy without renal transplantation or immunosuppressive drugs contribute to the development of infectious diseases and cancer.

For example, we can eliminate hepatitis C by direct acting antivirals. On contrary, regulation of epidemic virus infection is nowadays crucial, in context of covid-19, because we do not have any specific weapons against its pandemic. Covid-19 that anyone does not yet have immunity may be recognized as non-self pathogen and theoretically eliminated by NK cells [46]. As outcome of Covid-19 patients with preexisting CKD and HD is significantly poor [47], restoring the viral elimination mechanism of NK cells in uremic status may be effective in improving their prognosis.

## Conclusion and future directions

In this review, I described NK cell number, cytotoxicity, and activating mechanisms in the context of uremia and oxidative stress, particularly in ESKD patients undergoing HD. Due to differences in analytical methods, patient background, and changes in dialysis techniques over time, previous studies do not present a unified view.

Nevertheless, it is expected that the current knowledge of NK cell biology will inform clinical decision-making. As ROS could be a central actor in the modulation of NKG2D and MICA expression in ESKD [27], this provides a new rationale for the use of antioxidants or development of stress-free dialysis methods to maintain proper NK cell function. In contrast to the T- and B-cells involved in adaptive immunity, there is an incomplete understanding of the status of NK cell subsets in uremia [18, 48], and it would be beneficial to revisit innate immunity to viral infection and tumor development in ESKD for improved prognosis.

## Data Availability

Not applicable.

## Abbreviations

ESKD: End-stage kidney disease
NK: Natural killer
HD: Hemodialysis
NCR: Natural cytotoxic receptor
NKG2D: Natural killer group 2 member D
CD: Cluster of differentiation
NKp30, 44, 46: Natural killer cell p30-, 44-, 46-related protein
MHC: Major histocompatibility complex
MICA, MICB: MHC class I chain-related gene A, B
ITAM: Immunoreceptor tyrosine-based activation motif
CKD: Chronic kidney disease
ROS: Reactive oxygen species
